# Stealthy microbes: How *Neisseria gonorrhoeae* hijacks bulwarked iron during infection

**DOI:** 10.3389/fcimb.2022.1017348

**Published:** 2022-09-15

**Authors:** Julie Lynn Stoudenmire, Ashley Nicole Greenawalt, Cynthia Nau Cornelissen

**Affiliations:** Center for Translational Immunology, Institute for Biomedical Sciences, Georgia State University, Atlanta, GA, United States

**Keywords:** transferrin, hemoglobin, lactoferrin, *Neisseria gonorrhoeae*, iron, nutritional immunity, siderophore

## Abstract

Transition metals are essential for metalloprotein function among all domains of life. Humans utilize nutritional immunity to limit bacterial infections, employing metalloproteins such as hemoglobin, transferrin, and lactoferrin across a variety of physiological niches to sequester iron from invading bacteria. Consequently, some bacteria have evolved mechanisms to pirate the sequestered metals and thrive in these metal-restricted environments. *Neisseria gonorrhoeae*, the causative agent of the sexually transmitted infection gonorrhea, causes devastating disease worldwide and is an example of a bacterium capable of circumventing human nutritional immunity. *Via* production of specific outer-membrane metallotransporters, *N. gonorrhoeae* is capable of extracting iron directly from human innate immunity metalloproteins. This review focuses on the function and expression of each metalloprotein at gonococcal infection sites, as well as what is known about how the gonococcus accesses bound iron.

## Introduction

*Neisseria gonorrhoeae* (Ngo) is an obligate human pathogen responsible for the sexually-transmitted disease, gonorrhea (Unemo et al., 2019). Gonococcal infections are on the rise; in 2020, the World Health Organization (WHO) estimates an approximate 82.4 million people were newly infected with Ngo and the Centers for Disease Control and Prevention (CDC) reported 677,769 new cases in the United States (WHO, 2021; CDC, 2021). As antibiotic resistance increases, Ngo is a high priority for many agencies to monitor as an urgent threat pathogen (Ohnishi et al., 2011; WHO, 2021; Fifer et al., 2021). In December 2020 the CDC modified the recommended treatment of uncomplicated gonococcal infection, from dual therapy with ceftriaxone and azithromycin, to a higher dose of monotherapy ceftriaxone (Sancta St. Cyr et al., 2020). Prior infection does not provide protective immunity against reinfection and currently there is no effective vaccine, so at-risk individuals are often reinfected (Schmidt et al., 2001; Liu et al., 2011).

Ngo colonizes mucosal sites including the genital tract, rectum, conjunctiva, or oropharynx; genital infections often begin as urethritis in men and cervicitis in women (Schmidt et al., 2001; Walker and Sweet, 2011; Unemo et al., 2019). An estimated 80% of cases in women are asymptomatic, thus delaying treatment. Belated treatment may allow the infection to ascend the reproductive tract causing severe secondary sequalae in men and women (Portnoy et al., 1974; Walker and Sweet, 2011). Disseminated gonococcal infection (DGI) occurs when Ngo invades the bloodstream, sometimes due to delayed treatment; DGIs historically occur in less than 3% of cases, are more common in individuals less than 40, and occur more frequently in women than men (Rice, 2005; Walker and Sweet, 2011; Unemo et al., 2019; Li and Hatcher, 2020; Springer and Salen, 2020). In recent years, the numbers of DGI infections, particularly in men, have increased with no known link among cases (Belkacem et al., 2013).

**Figure 1:**
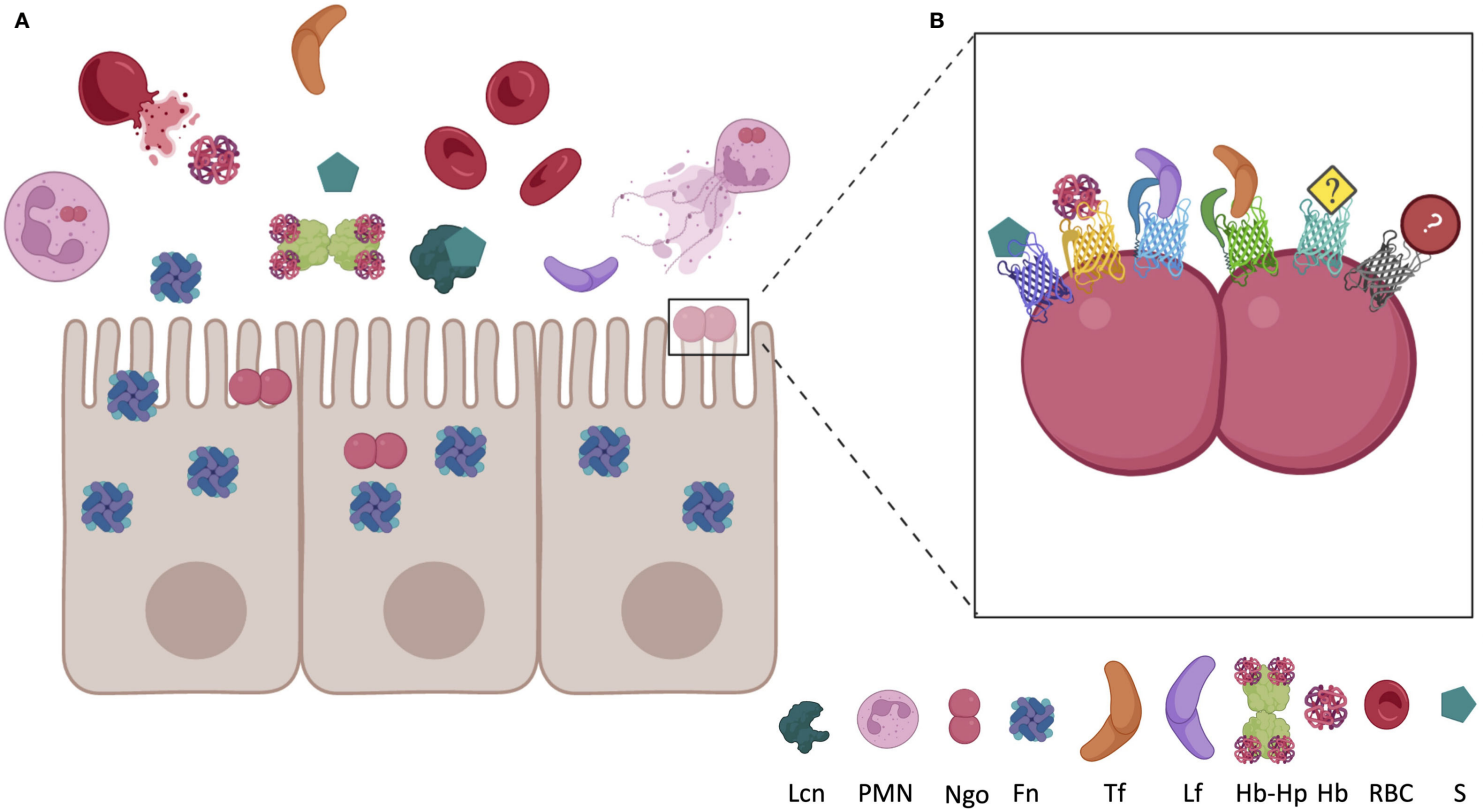
Localization of host nutritional immunity proteins near epithelial cell surface during inflammation and *Neisseria gonorrhoeae* (Ngo) infection. **(A)** In a healthy environment, iron is almost entirely bound to intracellular ferritin (Fn), erythrocyte bound haptoglobin, or sequestered in circulating transferrin (Tf). Under inflammatory conditions, Fn may be released from epithelial cells and hemoglobin (Hb) may be released from red blood cells (RBC). Haptoglobin (Hp) almost immediately binds to the newly circulating Hb, forming the Hb-Hp complex. Infection by Ngo recruits PMNs, which can expel their cellular contents in an innate immune response, which includes lactoferrin (Lf). Coinfection with other bacteria, or presence of commensals, may lead to circulation of siderophores (S). Siderophores produced by bacteria, or the mammalian siderophore 2,5-DHBA, may also be present at the site of infection bound to circulating lipocalin (Lcn). **(B)** Ngo has evolved mechanisms to cope with the nutritional immunity evoked by the host. TonB-dependent proteins bind many of the host Fe-chelating proteins, permitting Ngo to grow in these metal-restricted environments. FetA binds to S, HpuAB bind to Hb or Hb-Hp, TbpA binds to Tf, LbpA binds to Lf, and TdfF and TdfG are both iron regulated, but the host ligand has yet to be identified. Utilization of iron from ferritin and lipocalin should be investigated due to the close proximity to Ngo during infection. Figure not to scale and generated with BioRender.com.

Pathogens require metals for metabolism; therefore, there is a constant tug-of-war between host sequestration and pathogen acquisition for essential metals. Nutritional immunity is a host defense against infection where metalloproteins sequester essential nutrients away from pathogens (**Figure 1A**) (Hood and Skaar, 2012). Upon infection by Ngo, PMNs (Polymorphonuclear monocytes) are recruited to the site of infection, often forming NETs (Neutrophil Extracellular Traps), whereby the bacteria are exposed to the intracellular contents of the neutrophil, including several metal sequestration proteins [reviewed in (Criss and Seifert, 2012)]. Some Gram-negative pathogens have evolved ways to acquire iron directly from host metalloproteins, including transferrin (Tf), lactoferrin (Lf), and hemoglobin (Hb), using dedicated outer-membrane transporters [for a recent review see (Yadav et al., 2019)].

Access to, and availability of, metals in biological niches dictates the success and extent of infection by a pathogen. This review focuses on the roles of metalloproteins in regulating iron homeostasis in key gonococcal infection sites and how the gonococcus obtains the required iron for successful infection.

## Iron requirements and sequestration proteins in the human host

Iron is the most abundant metal in humans and is essential for metabolism in most aerobic organisms (Brock, 1999; Pantopoulos et al., 2012; Nairz et al., 2014; Golonka et al., 2019). During metabolism, iron acts as a cofactor in iron-sulfur (Fe-S) cluster proteins and heme-containing proteins, aiding in heme synthesis, oxygen transport, and DNA synthesis (Pantopoulos et al., 2012; Ganz and Nemeth, 2015). Iron is also important for proliferation of immune cells including T-lymphocytes and neutrophils (Brock, 1999; Weiss, 1999). Iron levels are stringently regulated in humans; iron overload is cytotoxic due to the generation of reactive oxygen species (ROS) and oxidative stress (Brock, 1999; Golonka et al., 2019). Hemochromatosis, or iron overload, can be caused by inherited genetic mutations, blood transfusions, or excessive dietary intake of iron, and may lead to increased susceptibility to infections and accelerated death (Khan et al., 2007; McDowell et al., 2022).

To prevent the toxic effects of free iron, over 99.9% of excess mammalian iron is sequestered intracellularly, either *via* ferritin or heme, and extracellular iron is bound to metalloproteins including Hb, Lf, and Tf (Pantopoulos et al., 2012; Andreini et al., 2018). Approximately 2% of the human genome encodes iron-containing proteins, of which, more than half of the proteins have a catalytic function (Andreini et al., 2018). Upon inflammation or infection by a pathogen, the liver secretes a peptide hormone, hepcidin, which modifies an iron exporter ferroportin, thereby trapping iron intracellularly (Nemeth et al., 2004b). By solubilizing iron, making iron bioavailable, chelating iron, and protecting the host from ROS, Fe-containing metalloproteins play essential roles in humans.

Hb, found within erythrocytes, is the most abundant protein in blood; Hb sequesters heme, which is a heterocyclic porphyrin ring that binds centrally-coordinated ferrous iron (Fe^2+^) (Baldwin, 1975). Hb is a globular protein consisting of α- and β-globulin chains, and inside erythrocytes, Hb stores approximately 75% of all the iron in the body and the remaining 25% is stored by ferritin in liver, spleen, and bone marrow (Brock, 1999; Delaby et al., 2005; Ganz and Nemeth, 2015). Hemoproteins, including hemopexin, Hb, and Hb complexed with haptoglobin (Hp), each bind heme strongly at one or two of the free iron-coordination sites located perpendicularly to the porphyrin ring (Hare, 2017). Erythrocytes spontaneously lyse, releasing up to 3 µM free Hb in healthy patients (Na et al., 2005). In serum, tetrameric Hb dissociates into dimers, which are rapidly sequestered by Hp, and the Hb-Hp complex is recycled by macrophages (Kristiansen et al., 2001). Hb may release heme spontaneously, particularly after oxidation to ferric Hb, or because of bacterial proteases (Na et al., 2005; Kassa et al., 2016; Hare, 2017).

Tf and Lf are glycoproteins of similar structure and function, sharing 60% sequence identity (Baker et al., 2002). Tf and Lf both contain a C-lobe and an N-lobe, with one Fe^3+^ ion bound to coordinating residues on each lobe (Aisen et al., 1978; Baker et al., 2002). Both Tf and Lf bind iron with nM affinity, and, notably, Lf maintains high affinity iron binding at low pH, down to pH 3.0, whereas Tf releases bound iron below pH 6.5 (Aisen et al., 1978; Baker and Baker, 2004).

Tf, at 80 kDa, is synthesized by hepatocytes and secreted into the serum where it solubilizes ferric iron, sequesters iron to prevent toxicity, and delivers iron into cells (Andrews and Ganz, 2019). Tf is naturally found at approximately 30% iron-saturation in serum (Ganz and Nemeth, 2015; Andrews and Ganz, 2019). While inflammation increases hepcidin concentrations, serum Tf concentrations decrease due to the decreased iron in circulation, causing a syndrome called anemia of infection (Ganz and Nemeth, 2009).

Lf, at 82 kDa is synthesized by neutrophils and exocrine glands and is primarily located in human milk and mucosal secretions (Masson and Heremans, 1968; Cohen et al., 1987; Kruzel et al., 2000; Rageh et al., 2016). Lf is antimicrobial and anti-inflammatory (Broekhuyse, 1974; Flanagan and Willcox, 2009; Okubo et al., 2016; Lepanto et al., 2019). Lf has been implicated as a regulator of inflammation (Baker and Baker, 2004; Alexander et al., 2012; Valenti et al., 2018). Lf is secreted by cervical and epithelial cells and found in secondary granules of human neutrophils (Lewis-Jones et al., 1985; Nuijens et al., 1992; Alexander et al., 2012; Valenti et al., 2018). Lf levels change in mucosal secretions at different stages of the menstrual cycle; Lf levels are lowest in the days before menstruation and highest proceeding menstruation when the cervix is more open, to prevent pathogenesis (Cohen et al., 1987). The fluctuation of Lf levels is likely hormone driven, as women taking oral contraceptives do not demonstrate an increase in Lf levels during menses, which could lead to higher infection rates (Cohen et al., 1987).

Humans produce siderocalins of the lipocalin family that chelate siderophores (Correnti and Strong, 2012; Sia et al., 2013; Page, 2019). Most Gram-negative bacteria produce siderophores, which scavenge environmental iron (Guerinot, 1994; Rohde and Dyer, 2003; Wandersman and Delepelaire, 2004; Miethke and Marahiel, 2007). Siderophores have such a high affinity and specificity for iron that they can pirate iron directly from Tf, Lf, but not heme (Raymond et al., 2003). By sequestering the bacterially produced siderophores, siderocalins can inhibit bacterial growth.

Lipocalin 2 (Lcn2) was first discovered as a neutrophil granule component and tightly binds bacterial catecholate ferric siderophores, including enterobactin; however, Lcn2 can also sequester some carboxylates (Kjeldsen et al., 2000; Goetz et al., 2002; Chakraborty et al., 2012). Mammalian catechols, often secreted in the urine, and the mammalian siderophore 2, 5-DHBA also bind to Lcn2; mammalian catechols may be derived from foods and 2,5-DHBA is produced from a gene with a bacterial homolog for the production of enterobactin (Bao et al., 2010; Devireddy et al., 2010). Lcn2 is produced by neutrophils, macrophages, hepatocytes, epithelial cells and adipocytes; therefore, it is present at mucosal sites at the initial stages of gonococcal infection and colonization (Kjeldsen et al., 2000; Chakraborty et al., 2012; Xiao et al., 2017).

## Acquisition of iron by *Neisseria*

TonB-dependent transporters (TDTs) are important for iron acquisition by Ngo and *Neisseria meningitidis*. TDTs are produced by most *Neisseria* strains and are highly conserved, suggesting TDTs play a significant survival role (Cornelissen et al., 1997a; Cornelissen et al., 2000; Cornelissen, 2008; Cornelissen and Hollander, 2011; Yadav et al., 2019). In Gram-negative bacteria, TDTs pirate iron, zinc, and other metals directly from host metalloproteins (Schryvers and Stojiljkovic, 1999; Cornelissen, 2018; Maurakis et al., 2019; Kammerman et al., 2020). TDTs are beta-barrels embedded in the outer membrane of the bacterium (Noinaj et al., 2013; Noinaj and Buchanan, 2014; Noinaj and Buchanan, 2018). With the help of TonB, TDTs extract metals, including iron and zinc, from host metalloproteins (Noinaj et al., 2012b; Cash et al., 2015; Maurakis et al., 2019; Kammerman et al., 2020).

The mechanism of metal import through TDTs is still being characterized. However, studies on TbpA suggest that a helical structure in the extracellular loops of the TDT may physically force the metal out upon binding of the ligand (Cash et al., 2015; Duran and Özbil, 2021). The extracted metal is immediately exposed to the plug domain of the TDT located in the pore of the beta-barrel, which may have a higher affinity for the metal than the ligand; thus, the metal ion relocates to the plug domain (Noto and Cornelissen, 2008). TonB is hypothesized to move the plug domain out of the barrel towards the periplasm, the metal ion is then exposed to a periplasmic binding protein that will ferry it to an ABC transporter, upon which the metal is imported into the cytoplasm, where it can then be used for essential metabolic processes, including replication within humans (Cornelissen et al., 1997b; Noinaj et al., 2010; Noinaj et al., 2012a; Noinaj et al., 2012b; Noinaj and Buchanan, 2014; Cash et al., 2015; Noinaj et al., 2017).

Several TDTs have been identified for their role in iron acquisition (**Table 1**; **Figure 1B**). Transferrin binding protein A (TbpA) is repressed by the ferric uptake regulator (Fur) under iron replete conditions (Agarwal et al., 2005). TbpA binds to hTf with an affinity of ~10 nM and is required for iron utilization from hTf (Cornelissen et al., 1992; Cornelissen and Sparling, 1996; Gray-Owen and Schryvers, 1996; Renauld-Mongénie et al., 2004; Noto and Cornelissen, 2008). Utilizing the human male model of gonococcal infection, a TbpAB knockout mutant was unable to establish an infection, suggesting essentiality of the system (Cornelissen et al., 1998). TbpA is a highly conserved 100 kDa, 22-stranded β-barrel outer-membrane receptor and TbpB is a more variable 85 kDa lipoprotein, which facilitates TbpA binding to iron loaded host Tf (Cornelissen and Sparling, 1996; Noinaj and Buchanan, 2014; Cash et al., 2015; Noinaj et al., 2017; Yadav et al., 2019). In Ngo, or *N. meningitidis* strains containing the type 2 variants of *tbpB*, TbpB is not essential for iron acquisition from Tf, but instead increases the rate iron uptake from hTf (Anderson et al., 1994; Renauld-Mongénie et al., 2004). *N. meningitidis* strains containing type 1 variants of *tbpB*, however, do require both proteins to bind hTf (Irwin et al., 1993). TbpA binds iron-saturated Tf or apo-Tf at similar rates (Tsai et al., 1988; Blanton et al., 1990; Anderson et al., 1994; Retzer et al., 1998).

**Table 1:** *Neisseria* express TonB-dependent transporters in response to iron limitation, which allow for the utilization of host nutritional immunity proteins as metal sources.

Neisseria gene(s)	Expression profile	Host protein	References
***tbpA/tbpB***	Fur-repressed	Human Transferrin	(Cornelissen et al., 1992; Agarwal et al., 2005)
***lbpA/lbpB***	Fur-repressed; found in approximately 50% of Ngo, 100% of *N. meningitidis*	Human Lactoferrin	(Schryvers and Morris, 1988; Biswas et al., 1999)
***hpuB/hpuA***	Fur-repressed; phase variable	Hemoglobin/hemoglobin:haptoglobin	(Lewis et al., 1997; Lewis et al., 1999)
***fetA***	Indirect Fur regulation; MpeR induced; phase variable	Bacterially produced siderophores	(Carson et al., 1999; Jackson et al., 2010; Hollander et al., 2011)
***tdfF***	Unknown regulation	Unknown	(Jackson et al., 2010)
***tdfG***	Unknown/potentially indirect Fur regulation	Unknown	(Jackson et al., 2010)

Lactoferrin-binding protein A (LbpA) binds to and extracts iron from human Lf (Schryvers and Morris, 1988; Pettersson et al., 1994; Biswas and Sparling, 1995; Anderson et al., 2003). LbpA is present in approximately 50% of gonococcal strains and all meningococcal strains and is Fur-repressed in high-iron environments and subjected to phase variation (Mickelsen and Sparling, 1981; Biswas and Sparling, 1995; Biswas et al., 1999; Anderson et al., 2003). Among the Ngo LbpA producers, only 30% express the lipoprotein LbpB, suggesting that LbpB is not required for Lf utilization (Bonnah and Schryvers, 1998; Biswas et al., 1999; Anderson et al., 2003; Cornelissen and Hollander, 2011). Similar to TbpB, LbpB binds primarily to holo-Lf (Yadav et al., 2021). While the presence of LbpAB increases competitive fitness over strains expressing the Tbp system alone, LbpAB is not essential for infection (Anderson et al., 2003).

Both TDTs TbpA and LbpA are capable of binding to, and extracting iron from, their human ligand in the absence of their respective lipoprotein partner; however, the TDT HpuB requires the lipoprotein HpuA to utilize the iron or heme from Hb and Hb-Hp complexes (Lewis et al., 1997; Lewis et al., 1998; Lewis et al., 1999; Rohde et al., 2002; Rohde and Dyer, 2004). HpuB (85 kDa) is the outer-membrane receptor (Postle, 1993; Klebba et al., 1993) and HpuA (35 kDa) is the lipoprotein partner (Lewis et al., 1997; Lewis et al., 1999; Rohde et al., 2002; Anzaldi and Skaar, 2010). In *N. meningitidis*, HpuAB binds to Hb, Hb-Hp, and apo-haptoglobin (Lewis et al., 1999). *hpuAB* undergoes phase variation due to slipped-strand mispairing, resulting in a frameshifted non-functional protein (Lewis et al., 1997; Chen et al., 1998; Lewis et al., 1999). Further, *hpuAB* is Fur repressed under iron replete conditions. (Lewis et al., 1997). Gonococcal isolates collected from women in the first two weeks of their menstrual cycle are more likely to express HpuAB, suggesting that when Hb and Hp are abundant, Ngo producing HpuAB is under selective pressure to be expressed (Chen et al., 1998; Anderson et al., 2001).

Ngo is unable to synthesize siderophores; however, the gonococcus can use siderophores produced by other bacteria, including salmochelin, enterobactin, and dihydroxybenzoylserine acid through the TDT FetA (West and Sparling, 1987; Carson et al., 1999; Strange et al., 2011). FetA is an 80 kDa outer membrane transporter that is iron repressed and induced by MpeR, an AraC-like regulator, under iron-deplete conditions (Hollander et al., 2011). FetA is phase variable *via* slipped-strand mispairing (Carson et al., 1999). Additionally, MpeR is regulated by Fur and is pathogen specific, suggesting FetA is potentially upregulated as a virulence factor under iron limiting conditions (Snyder and Saunders, 2006; Marri et al., 2010; Jackson et al., 2010).

Repressed under iron replete conditions, both TDTs, TdfF and TdfG, have been implicated in iron acquisition by Ngo. TdfF, an 80 kDa outer membrane protein, is produced exclusively by the pathogenic *Neisseria*, which could suggest importance as a virulence factor (Turner et al., 2001). While no ligand has been identified to interact with TdfF, in some strains of Ngo, TdfF does contribute to intracellular survival in a TonB-dependent way (Hagen and Cornelissen, 2006). Utilizing the FA1090 Ngo sequence for bioinformatic analysis, the largest of the TDTs at 136 kDa, TdfG is exclusive to Ngo and *Neisseria elongota* (Turner et al., 2001; Marri et al., 2010). Like TdfF, no ligand has been identified for TdfG and little more is known about how TdfG contributes to Ngo growth or survival in humans. Thus far, little is known about the regulation of gene expression for either TdfF or TdfG, though a Fur-independent mechanism has been proposed for TdfG regulation (Jackson et al., 2010).

## Host iron cycling: Infection and inflammation

Bacterial infection and inflammation act as signals for the host to deplete iron by activating an acute phase response and/or upregulating nutrient sequestration mechanisms (Weinberg, 1975; Ganz and Nemeth, 2015; Cornelissen, 2018; Golonka et al., 2019). Low blood iron during the first 24 hours of infection in patients was first described in the 1940s (Cartwright et al., 1946). Cytokines and tissue damage from inflammation are known to induce hepcidin production in the liver, promoting iron, heme, and Hb sequestration by macrophages and other iron-storage cells (Nemeth et al., 2004a; Armitage et al., 2011; Ganz and Nemeth, 2015; Ross, 2017). As serum iron levels dip below physiological levels of 10 to 30 µM, erythropoiesis, or the synthesis of erythrocytes, is inhibited freeing the iron for other processes (Ganz and Nemeth, 2015).

Ngo can invade cells, including macrophages and neutrophils which are the first immune cells to arrive at the site of infection (Zughaier et al., 2014). Iron retention in macrophages could be particularly beneficial for gonococcal infection, as iron retention in macrophages inhibits nitric oxide formation which aids in killing of intracellular bacteria (Nairz et al., 2014). Interestingly, upon infection of monocytes and macrophages, Ngo can upregulate hepcidin and downregulate ferroportin, resulting in an overall increase of iron retention (Zughaier et al., 2014). Ngo and *N. meningitidis* reduce expression of the host transferrin receptor in infected epithelial cells (Bonnah et al., 2000; Bonnah et al., 2004). The gene expression profiles of gonococcal or meningococcal infected cells mimic cells propagated in a low-iron environment, suggesting infection of these cells either shuttles all available iron to the infecting pathogens, generating a low-iron environment for the eukaryotic cells, or a signal from the pathogens may alter the regulatory network (Bonnah et al., 2004).

## Perspectives: Potential pathways for treatment and prevention

TDTs have been suggested as vaccine candidates because they are highly conserved, present in pathogenic *Neisseria*, and most are not subject to high-frequency antigenic variation (Cornelissen et al., 2000; Cornelissen, 2008; Martinez-Martinez et al., 2011; Noinaj et al., 2012a; Cash et al., 2015; Frandoloso et al., 2015; Martínez-Martínez et al., 2016; Martinez-Martinez et al., 2016; Rice et al., 2017; Chan et al., 2018; Russell et al., 2019). This review summarizes the important iron metalloproteins and tissue specialization involved in neisserial pathogenesis. TbpA is essential for infection; LbpA, aids in pathogenesis; HpuAB is upregulated in females during the first half of their menstrual cycle; and TdfF is essential for intracellular survival. Consequently, these iron-regulated TDTs are also attractive targets for future therapeutics.

*Neisseria* species have the ability to capitalize on many mammalian nutritional immunity tactics by utilizing the iron from these chelating proteins. TbpA and LbpA bind only the human versions of transferrin and lactoferrin, respectively, suggesting a tightly co-evolved system of nutrient acquisition. Some potential iron sinks have not been assessed for their ability to support neisserial growth. For example, no evidence is available on whether *Neisseria* are capable of exploiting Lcn2, ferritin, or NRAMP-1, all of which are upregulated at infection sites in response to infection/inflammation. Human calprotectin is found in high concentrations in PMNs, and recently, calprotectin has been described as binding iron with high affinity (Urban et al., 2009; Nakashige et al., 2015). Further, the TDT, TdfH, binds to and utilizes the Zn bound to calprotectin (Jean et al., 2016). Thus, calprotectin is in close proximity to Ngo during infection and the interaction between calprotectin and Ngo has been described; however, calprotectin binds Fe(II) with high affinity, whereas all known Ngo iron sources are Fe(III), making calprotectin an unlikely source of iron for Ngo (Nakashige et al., 2015). It is possible that TDTs can bind to and utilize metals from multiple iron sources, thus it is important to assess potential metal sources in an unbiased way.

## Author contributions

JS and AG completed the literature review and manuscript drafting. JS edited based on comments by CC. CC reviewed and proofread the manuscript and acquired funding. All authors contributed to the article and approved the submitted version.

## Funding

This work was funded by the National Institute of Allergy and Infectious Diseases, award numbers U19AI144182, R01AI127793, and R01AI125421 to CC. The funder had no role in data collection, synthesis, analysis, interpretation, or management of the data presented in this review. The funder had no role in review generation and revision, or in the decision to submit this review for publication.

## Acknowledgments

We thank the support from the Institute for Biomedical Sciences at Georgia State University.

## Conflict of interest

The authors declare that the research was conducted in the absence of any commercial or financial relationships that could be construed as a conflict of interest.

## Publisher’s note

All claims expressed in this article are solely those of the authors and do not necessarily represent those of their affiliated organizations, or those of the publisher, the editors and the reviewers. Any product that may be evaluated in this article, or claim that may be made by its manufacturer, is not guaranteed or endorsed by the publisher.
